# The Hard Road to Greatness

**Published:** 1881-02

**Authors:** 


### THE HARD ROAD TO GREATNESS.

A person who has achieved distinction in science, literature, art or politics is generally credited with the possession of great mental powers. Those who stand near the head of their respective professions are popularly supposed to owe their eminence to that vague, indefinable attribute which we call genius. Thousands of men look up in profound admiration and solemn awe to the height which some old acquaintance has attained, attributing his success to some phenomenal mental endowment, when, as a matter of fact, each of his admirers might have reached the same altitude by pursuing the same policy he adopted, and making the same effort he has made.

Greatness is within the reach of the average child. Any healthy boy or girl who will make early selection of the proper line of work, and faithfully devote a life thereto, can achieve distinction—can get on top, or very near it. It will be a life of toil and sacrifice. Its devotee will have little opportunity for domestic enjoyment or social pleasures. He must find in his work a substitute for every other source of pleasure.

The man who increases the sum total of successful knowledge by so much as a single fact, has made his fame secure. He who excels the best of his predecessors in any one of ten thousands lines of effort, has written his name in the catalogue of the illustrious. And as each new student has the work of all his predecessors before him, and can begin his searchings for the attainable at the point where the most advanced of them stopped, it requires only work—and enough of it—to carry the frontier of progress a step further on, to push the invasion of the unknown a degree further than any previous explorer, to pick up a few pebbles of knowledge on the shores of the boundless sea, at a point never before reached.—*Hebrew Leader.*

### CRUEL DREAMS.

I fall asleep:
Then he arrives and whispers in my ear,
"The past is not. He whom you love is here;
No longer weep!"

"I am not dead,"
He says, and takes me gently by the hands,
And leads me to those pleasant yellow sands
We used to tread.

He softly talks
Of all the things we talked of long ago;
And I am happy, pacing to and fro
Those well-loved walks!

But when I try
To tell of what had happened since the day
He went, ah me, he slowly fades away!
I wake—and cry.

—*London World.*

